# Severe Extrahematopoietic Manifestations in Complete STAT1 LOF after Successful Allogeneic HCT

**DOI:** 10.1007/s10875-024-01789-4

**Published:** 2024-09-04

**Authors:** Friederike Frieß, Michael Flaig, Michael H. Albert, Christoph Klein, Fabian Hauck

**Affiliations:** 1 Department of Pediatrics, Dr. von Hauner Children’s Hospital, University Hospital, Ludwig-Maximilians-Universität München, Lindwurmstrasse 4, D-80337 Munich, Germany; 2 Department of Dermatology and Allergy, University Hospital, Ludwig-Maximilians-Universität München, Munich, Germany

## To the Editor

Inborn errors of human signal transducer and activator of transcription 1 (STAT1) lead to a broad spectrum of immunodeficiency with susceptibility to infections and immunodysregulation with autoinflammation and autoimmunity. STAT1 mediates cellular responses to interferon-α/β and interferon-γ receptor binding through nuclear translocation and transcriptional activation of target gene expression inducing antimicrobial and inflammatory responses. [1] Complete autosomal recessive (AR) loss-of-function (LOF) mutations lead to the absence of STAT1 expression and function. [1, 2] Patients suffer from both severe viral and mycobacterial infections at an early age resulting in poor prognosis. [1] AR STAT1 deficient patients show an almost complete loss of induction of a broad range of interferon-stimulated genes after IFN-α2b, IFN-λ, IFN-γ and IL-27 stimulation. [3] Patients with AR STAT1 deficiency have been described to develop episodes of hemophagocytic lymphohistiocytosis (HLH) secondary to live vaccines, other infectious pathogens or with no identifiable trigger. [3, 4] The underlying mechanisms responsible for hyperinflammation are not yet fully understood. With limited therapeutic options early correction of hematopoietic STAT1 LOF through allogeneic hematopoietic cell transplantation (HCT) is promising. Eleven patients with complete AR mutations who underwent HCT have been reported. [3, 4] Four patients died due to viral and mycobacterial infections in up to 96 days after HCT. [3] The seven surviving patients were not reported to develop severe viral or mycobacterial infections after engraftment with a median follow-up of 45.7 months. [3]

## Case Details

**Fig. 1:**
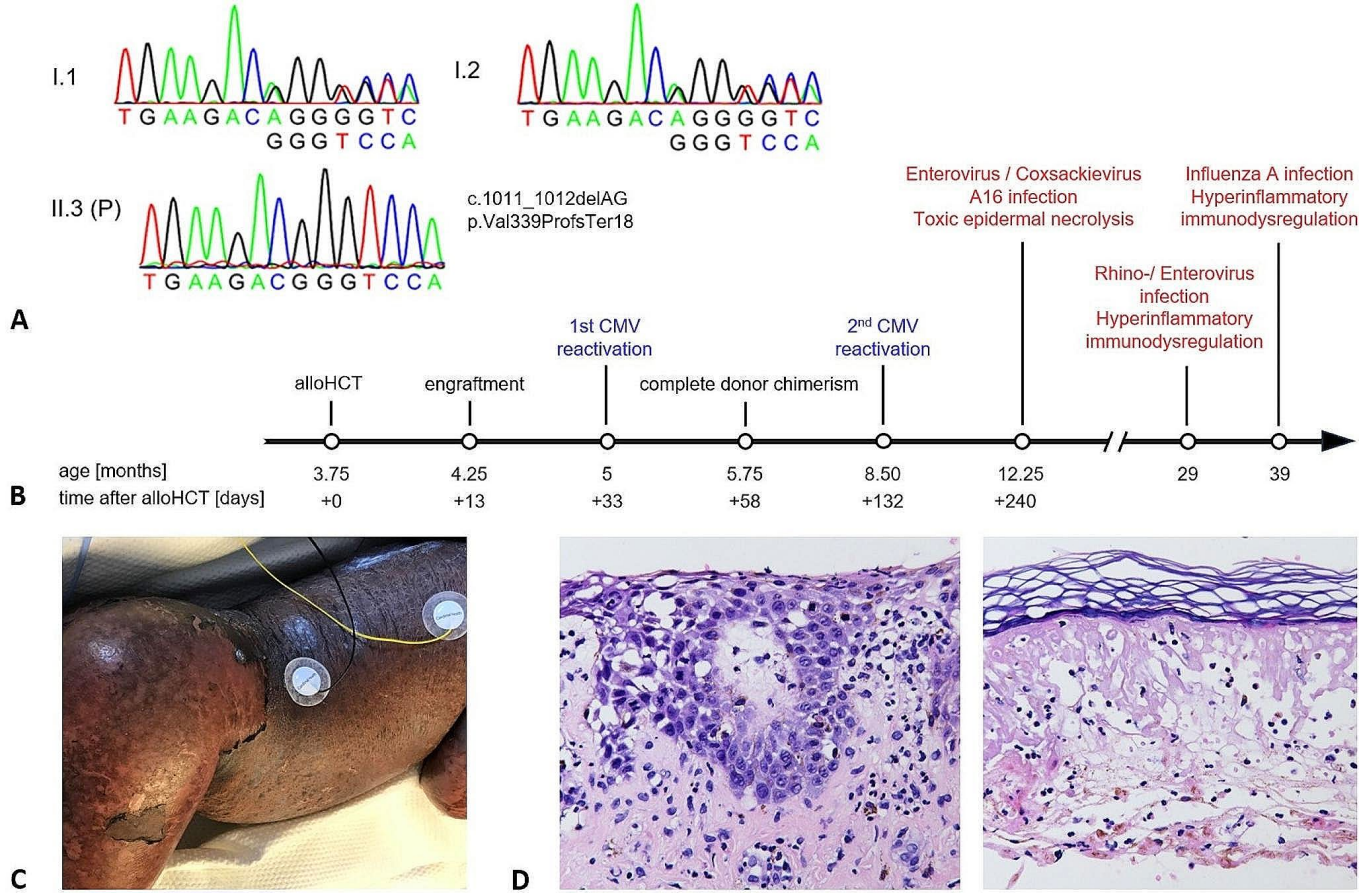
**(A)** Electropherogram of parents and patient (P). **(B)** Timeline of the patient´s clinical course. **(C)** Enterovirus/Coxsackievirus A16 infection leading to epidermolysis at the age of 12.25 months. **(D)** Histopathology of patient´s skin showing degenerative interface dermatitis and single cell necrosis on the base of the blister and complete necrosis with eosinophilic and neutrophilic granulocytic infiltration on the roof of the blister. *AlloHCT*, allogeneic hematopoietic cell transplantation. *CMV*, cytomegalovirus. *STAT1*, signal transducer and activator of transcription 1

In contrast, we report a 3.7-year-old girl with prenatal diagnosis of complete AR STAT1 LOF (homozygous STAT1 c.1011_1012delAG; p.Val339ProfsTer18) (Fig. 1A) with development of atypical disease patterns triggered by viral infections after early and successful allogeneic HCT (Fig. 1B). The patient is the third child from healthy consanguineous parents of West African origin. The prenatal diagnosis was made due to her older brother who was diagnosed with complete AR STAT1 LOF by identifying a novel homozygous STAT1 frameshift variant after a clinical history of severe viral infections. The parents were identified to be heterozygous carriers (Fig. 1A). [4] After birth the patient was put into protective isolation and received antimicrobial prophylaxis using cotrimoxazole, azithromycin, nystatin, and IV immunoglobins (IVIG) every four weeks. The conditioning regimen consisted of fludarabine (40 mg/m^2^/d on days -7 to -4), busulfan (4.8 mg/kg/d on days -7 to -4; targeted area under the curve 87.876 ng*h/ml) and anti-thymocyte globulin (Grafalon^®^; 10 mg/kg/d on days -4 to -3). Allogeneic HCT was performed at the age of 15 weeks using 6.9 × 10^8^/kg nucleated cells of erythrocyte depleted marrow from her HLA-identical father (5.8 × 10^6^ CD34^+^/kg, 74.1 × 10^6^ CD3^+^/kg). Engraftment was observed on day +13. Under GvHD prophylaxis using cyclosporine A and mycophenolate mofetil there was no development of acute GvHD. At the age of four months (day +33) the patient was admitted to hospital for treatment of CMV reactivation. The patient received IV ganciclovir, CMV hyperimmunoglobulin and letermovir. The patient received valganciclovir prophylaxis until day +120 when immunosuppression was also discontinued. On day +58 complete donor chimerism was observed in peripheral blood (Supplementary Table S1). On day +132 the patient again presented with CMV reactivation treated with IV ganciclovir, and valganciclovir prophylaxis was reestablished. On day +180 chimerism and immune reconstitution were complete (Supplementary Table S1). One year after allogeneic HCT phosphorylation of STAT1 in PBMCs was tested, showing normal STAT1 expression and phosphorylation upon interferon stimulation in CD14 monocytes (Supplementary Figure S1) machting simultaneous chimerism testing. On day +240 the patient was readmitted presenting with fever and vesicular exanthema. The exanthema spread from the head to the whole body including the oral mucosa and the conjunctivae followed by the development of bullae (Fig. 1C). Enterovirus/Coxsackievirus A16, that has a tropism for keratinocytes and is a known cause of hand, foot, and mouth disease in immunocompetent individuals, was isolated from cerebrospinal fluid, blood, stool, and skin samples. The affected epidermis nearly completely detached in up to 10% body surface. Histopathology showed toxic epidermal necrolysis with inflammation and edema (Fig. 1D). Dermatologists diagnosed Stevens-Johnson syndrome (SJS) and/or toxic epidermal necrolysis (TEN). The critically ill patient with epidermolysis, hypovolemic renal failure, and hepatitis was treated on the PICU like a severe burn. Under local treatment with dexpanthenol and polihexanide she recovered over a period of eight days with scar free healing. 29 months after HCT, the patient was readmitted to hospital presenting with fever, abdominal pain and a papulous exanthema on the hands, inguinal region, and feet. A throat swab multiplex PCR detected human Rhino-/Enterovirus. The patient developed vomiting, diarrhea, and recurring fever unresponsive to antibiotics. Acute-phase proteins were elevated with C-reactive protein 14.6 mg/dl (reference range < 0.8), procalcitonin 0.3 ng/ml (< 0.1) and fibrinogen 702 mg/dl (160–400) without signs of HLH. Assuming hyperinflammatory immunodysregulation, IVIG 2 g/kg and methylprednisolone 2 mg/kg/d were administered, and the skin rash and fevers resolved. From this time onwards the patient´s chimerism showed to be mixed, but stable over time until 2.9 years after allogeneic HCT (Supplementary Table S1). Regularly performed immunophenotyping of peripheral blood of the patient showed stable numbers within the reference ranges (Supplementary Table S1). At the age of 39 months the patient was again admitted to hospital presenting with pneumonia with need for oxygen supplementation testing positive for influenza A virus. Laboratory abnormalities showed a HLH-like hyperinflammatory immunodysregulation (ferritin > 8000 ng/ml (10–500), triglycerides 372 mg/dl (25–119), thrombocytes 22 G/l (195–464), hemoglobin 10.2 g/dl (10.7–13.4), GPT 1374 U/l (< 59), fibrinogen 94 mg/dl (160–400), sCD25 5810 U/ml (158–623)). Steroid treatment was initiated (prednisolone 1.3 mg/kg/d). Within one week the respiratory distress resolved and the inflammatory parameters normalized.

## Discussion

This is the first patient with AR STAT1 LOF to be reported to suffer from severe viral infection-triggered immunopathology such as toxic epidermal necrolysis (TEN) and hyperinflammatory immunodysregulation after successful allogeneic HCT. Other surviving patients with complete AR STAT1 LOF were not reported to display comparable features after engraftment with a median follow-up time of 45.7 months after HCT. [3] Two patients developed progressive lunge disease two and four years after allogeneic HCT. [3] The patient´s brother showed a defective control of VSV in monocytes and fibroblasts in association with increased proinflammatory cytokine production. [4] Boehmer et al. demonstrated that complete AR STAT1 LOF increases the susceptibility to viral infections with an increased viral replication compared to healthy controls leading to production of proinflammatory cytokines such as IL-6, TNF, and IL-18 which can result in hyperinflammation. [4] Examination of severe viral infections in otherwise healthy patients revealed specific mutations leading to a disruption of organ specific cell-intrinsic immunity. [5] Disruptions of type I and III interferon signaling in plasmacytoid dendritic cells and pulmonary epithelial cells have been reported to cause severe influenza A pneumonitis in patients with AD GATA2, AR IRF7, and IRF9 deficiencies, respectively. [5] Mutations in TLR3 were found to disrupt interferon-β and -γ responses in neuron- and oligodendrocyte-intrinsic immunity leading to forebrain HSV1 encephalitis. [5] These observations support the assumption that cell-intrinsic immunity of non-hematopoietic cells can be critical for viral disease control. In the case of AR STAT1 LOF reported here, despite complete hematopoietic donor chimerism until 2.4 years after allogeneic HCT, extrahematopoietic cells remained vulnerable towards viral infections. The following mixed chimerism showed stable numbers of donor cells in the patient´s peripheral blood ensuring a recovered STAT1 function in hematopoietic cells (Supplementary Table S1). The combination of extrahematopoietic complete STAT1 deficiency with complete hematopoietic STAT1 corrections, lead to unprecedented diseases patterns triggered by viral infections and putatively aggravated by STAT-signaling imbalances and STAT1-proficient adaptive immune pathology. This phenotype can only be observed in human chimera and points towards potential extrahematopoietic somatic variants of intrinsic and innate immunity as putative drivers of more common organ-specific severe viral infections and/or inflammation in otherwise healthy individuals.

## Electronic Supplementary Material

Below is the link to the electronic supplementary material.

Supplementary Material 1

## Data Availability

No datasets were generated or analysed during the current study.

## Statements and Declarations

This work was supported by the Care-for-Rare Foundation (160073) and the German Federal Ministry of Education and Research (BMBF) - German genetic multi-organ Auto-Immunity Network (GAIN) (01GM2206B).
