# Supplementary material for: Severe Extrahematopoietic Manifestations in Complete STAT1 LOF after Successful Allogeneic HCT
Source: J Clin Immunol. 2024 Sep 4;44(8):189. doi: 10.1007/s10875-024-01789-4 (PMC11374845; doi:10.1007/s10875-024-01789-4)
Supplement: Supplementary file 1 — Supplementary Material 1 [file 10875_2024_1789_MOESM1_ESM.docx]

Journal of Clinical Immunology

**Severe extrahematopoietic manifestations in complete STAT1 LOF after successful allogeneic HCT**

Friederike Ehring, MD^1^, Michael Flaig, MD^2^, Michael H. Albert, MD^1^, Christoph Klein, MD, PhD^1^, Fabian Hauck, MD, PhD^1,*^

^1^Department of Pediatrics, Dr. von Hauner Children's Hospital, University Hospital, Ludwig-Maximilians-Universität München, Munich, Germany.

^2^Department of Dermatology and Allergy, University Hospital, Ludwig-Maximilians-Universität München, Munich, Germany.

*Corresponding author

Fabian Hauck, MD, PhD

Dr. von Hauner Children’s Hospital, University Hospital Munich

Ludwig-Maximilians-Universität München

Lindwurmstrasse 4

D-80337 München

Email: [fabian.hauck@med.uni-muenchen.de](mailto:fabian.hauck@med.uni-muenchen.de)

Phone: +49(0)89440053931

Fax: +49(0)89440053964

**Supplemental Information**

**TABLE S1.** Patient´s chimerism and immune reconstitution over time after hematopoietic cell transplantation (HCT). Chimerism measured in patient´s peripheral blood using fluorescence in situ hybridization (FISH) marking female (patient) and male (donor) interphase nuclei (CEP X/CEP Y, Co. Abbott). Specific chimerism for T cells (CD3+) and B cells (CD19+) measured by FISH after separation using CD3 and CD19 antibodies. Immunophenotyping of T cells (CD3+), B cells (CD 19+) and HLA-DR+ cells measured in peripheral blood of the patient.

| Time after HCT (years) | Chimerism (female/male interphase nuclei) | CD3+ cells/μl (reference range) | CD19+ cells/μl (reference range) | HLA-DR+ T cells, B cells, Monocytes/μl (reference range) |
| --- | --- | --- | --- | --- |
| 0.3 | Complete | 1383 (2400-6900) | 381 (700-2500) | 1503 (770-3000) |
| 1.0 | Complete | 3616 (1400-8000) | 723 (600-3100) | 2531 (700 – 3800) |
| 2.4 | Mixed  Global (45/102) | 4296 (900-4500) | 1392 (200-2100) | 3328 (280-2500) |
| 2.6 | Mixed  Global (42/101)  T cells (11/113) | 3582 (900-4500) | 619 (200-2100) | 2211 (280-2500) |
| 2.9 | Mixed  Global (62/112)  T cells (11/115)  B cells (20/105) | 1116 (900-4500) | 326 (200-2100) | 667 (280-2500) |


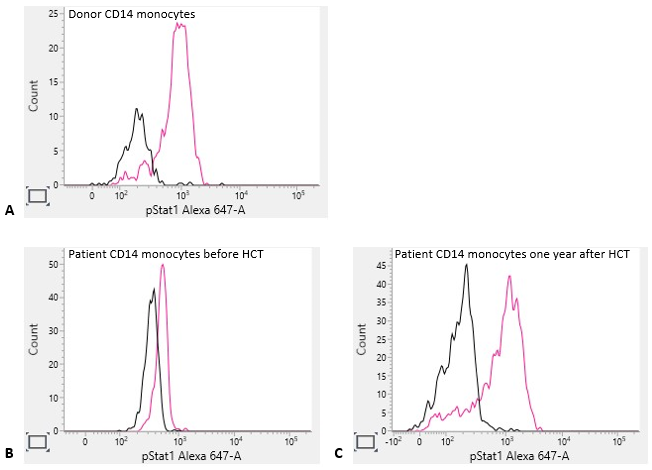


**FIGURE S1.** Flow cytometry histograms of anti-phosphorylated STAT1 intracellular binding in CD14 monocytes collected from peripheral blood before (black line) and after (purple line) interferon-gamma stimulation for **(A)** donor CD14 monocytes, **(B)** patient CD14 monocytes before hematopoietic cell transplantation (HCT) and **(C)** patient CD14 monocytes one year after hematopoietic cell transplantation.
